# Determinants of the use of insecticide-treated mosquito nets in pregnant women: a mixed-methods study in Ghana

**DOI:** 10.1093/inthealth/ihab087

**Published:** 2022-01-22

**Authors:** F Dun-Dery, N Kuunibe, P Meissner, V Winkler, A Jahn, O Müller

**Affiliations:** Ghana Public Health Association; Heidelberg Institute of Global Health, Medical School, Ruprecht-Karls-University Heidelberg, Im Neuenheimer Feld 130.3 Heidelberg, Germany; Simon Diedong Dombo University of Business and Integrated Development Studies, Wa, Ghana; Pediatric Clinic, Klinikum Konstanz, Mainaustraße 35, 78464 Konstanz, Germany; Heidelberg Institute of Global Health, Medical School, Ruprecht-Karls-University Heidelberg, Im Neuenheimer Feld 130.3 Heidelberg, Germany; Heidelberg Institute of Global Health, Medical School, Ruprecht-Karls-University Heidelberg, Im Neuenheimer Feld 130.3 Heidelberg, Germany; Heidelberg Institute of Global Health, Medical School, Ruprecht-Karls-University Heidelberg, Im Neuenheimer Feld 130.3 Heidelberg, Germany

**Keywords:** insecticide-treated mosquito nets, knowledge, malaria complications, mixed methods, pregnant women

## Abstract

**Background:**

Malaria in pregnancy remains a significant cause of morbidity and mortality, affecting the highly endemic countries of sub-Saharan Africa (SSA). Insecticide-treated nets (ITNs) are effective for malaria prevention. However, poor adherence in SSA remains a challenge.

**Methods:**

We conducted a standard questionnaire survey among 710 pregnant women from 37 primary care clinics in the Upper West Region of Ghana from January through May 2019. Using a sequential explanatory design, we integrated the survey data from six focus group discussions with pregnant women.

**Results:**

While 67% of women had some general knowledge about malaria prevention, only 19% knew the specific risks in pregnancy. Determinants of ITN use included ITN ownership (odds ratio [OR] 2.4 [95% confidence interval {CI} 1.3 to 4.4]), good maternal knowledge of the risks of malaria in pregnancy (OR 2.4 [95% CI 1.3 to 4.3]) and more antenatal care (ANC) contacts (OR 1.3 [95% CI 1.0 to 1.5)]. Focus group discussions showed that non-use of ITNs resulted from inappropriate hanging infrastructure, a preference for other malaria prevention alternatives, allergy and heat.

**Conclusions:**

Specific maternal knowledge of malaria risks in pregnancy was low and influenced the regular use of ITNs. Community and ANC-based malaria interventions should prioritize increasing knowledge of the specific risks of malaria.

## Introduction

Despite the remarkable global efforts in the fight against malaria, the disease has remained a huge burden for pregnant women. It often compromises the mother's health and puts her at greater risk of death. Malaria in pregnancy impacts the health of the foetus, often leading to prematurity and low birthweight (LBW), which are significant contributors to neonatal and infant mortality.^1^ Approximately 25–30 million women get pregnant in sub-Saharan Africa (SSA) each year; about 11 million are exposed to malaria, 900 000 deliver a child with LBW and 10 000 mothers die.^1–4^ To protect pregnant women in SSA, the World Health Organization (WHO) recommends using insecticide-treated nets (ITNs) in addition to preventive antimalarial medicines.^5^ When properly used, ITNs can reduce malaria transmission by at least 60%.^6^ However, 40% of pregnant women globally did not use ITNs in 2018.^1^ A number of studies conducted across malaria-endemic areas of SSA regarding knowledge of malaria control measures among pregnant women indicate that knowledge of malaria risks during pregnancy is relatively high.^3^

In Ghana, 7 in 10 people in the general population had access to an ITN from the latest mass distribution campaign in 2018.^7^ Specifically among pregnant women, ownership of ITNs nationwide increased slightly, from 32.8% in 2015 to about 41% in 2016,^8^ but only about half of the 41% who owned ITNs used them during this period.^9–11^

Anaemia (haemoglobin <11.0 g/dL) in pregnancy was 56.0% in 2019,^12^ and malaria accounted for 18.0–33.0% of overall outpatient hospital attendance,^12^ and 11–34.3% of overall hospital admissions.^7^ There is ample evidence that maternal knowledge of malaria risks in pregnancy plays a significant role in malaria prevention.^13^ However, it is unknown if this preventable malaria burden is caused by a lack of knowledge, a lack of prevention tools or both. This study investigated the relationship between maternal malaria knowledge and ITN use among pregnant women in Ghana.

## Methods

### Study area and population

**Figure 1.**
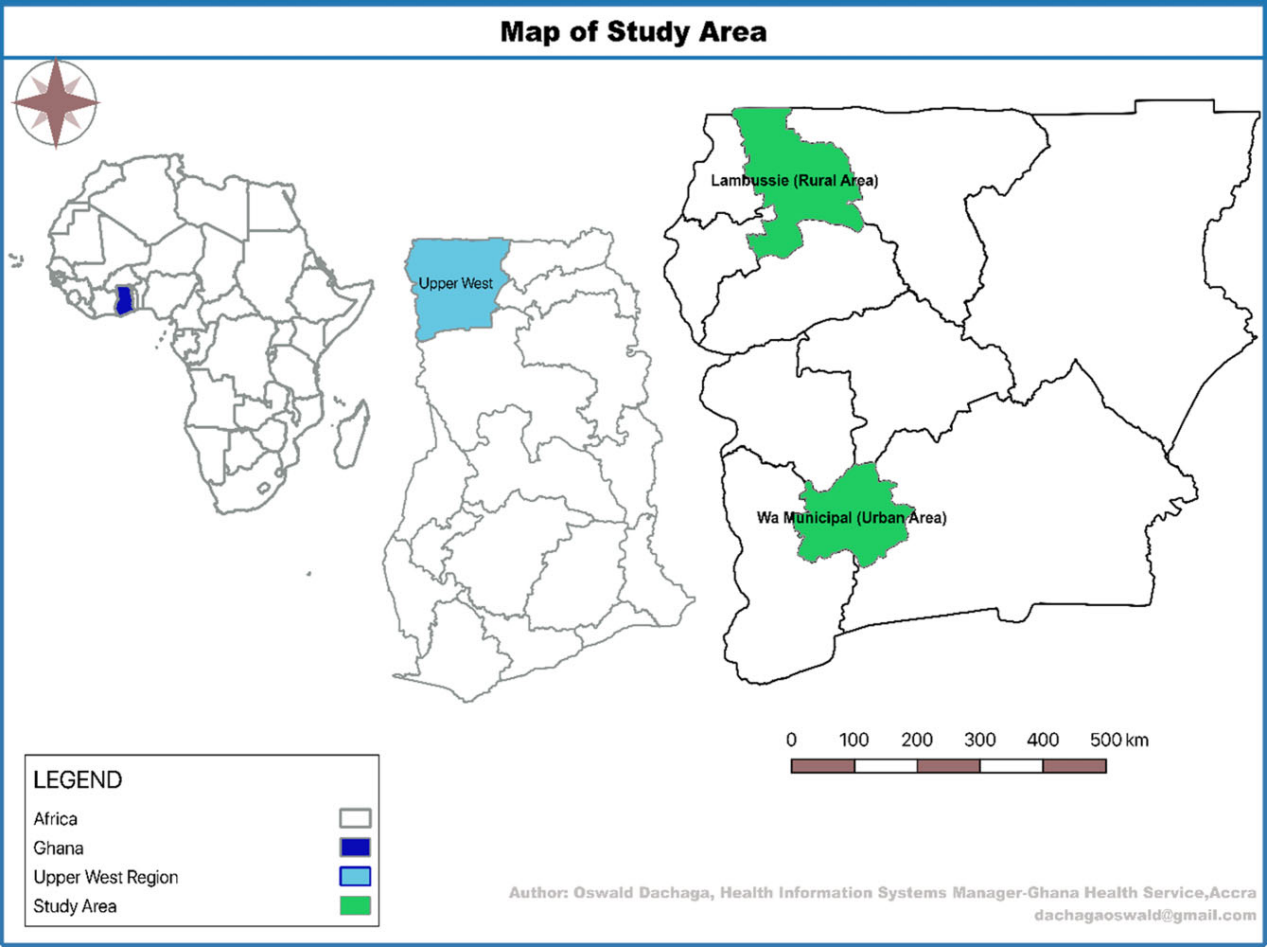
Map of the study area (Upper West Region), showing the two districts selected.

The study was conducted in the Upper West Region (UWR) of Ghana (population 868 484 in 2020), an area that is highly endemic for malaria (Figure 1).^14^

### Definition of terms

#### ITN use

ITN use was defined as a pregnant woman having slept under an ITN the night previous to the study interview.

#### General maternal knowledge of malaria

A pregnant woman was considered to have good general knowledge regarding the transmission and prevention of malaria if her average score for the knowledge-based questions was at least 50%.

#### Specific maternal knowledge of risks of malaria

A pregnant woman was considered to have good specific knowledge regarding the risks that can result from malaria infection during pregnancy if she listed at least two-thirds of these specific risks correctly.

### Study design

A comparative sequential explanatory mixed-methods model was used.^15^ First, a survey was conducted among third-trimester pregnant women attending antenatal care (ANC) services in two districts of the UWR. The study focused on third-trimester pregnant women because, per Ghana's free maternal healthcare policy, ITNs are freely issued to pregnant women upon registration of their pregnancies at the health facility (HF). We presumed that third-trimester women may have had the optimum number of ANC contacts (≥4) and would have been exposed to regular facility-based ANC education on malaria. In addition, focus group discussions (FGDs) were conducted with pregnant women in the area. The study took place from January to May 2019.

### Quantitative data

Quantitative data were collected by administering a standard questionnaire, review of respondents’ ANC records and direct observations. These data included respondents’ sociodemographic and obstetric characteristics and their use or non-use of ITNs. We also assessed the pregnant women's knowledge of malaria transmission, the risk to pregnancy and malaria prevention. A mean score was calculated for general knowledge using only correct responses, while the specific knowledge of malaria risks in pregnancy was used in the logistic regression model. With an α level of 5% (two-sided t-test) and a power of 80%, the total estimated sample size (N=n_1_+n_2_) needed to detect any variations by comparing the two study districts was 710 (n_1_=355) third-trimester pregnant women, considering the proportions of ITN use in the rural and urban districts to be 70% and 60%, respectively, as described in similar populations.^16,17^

### Selection of study districts and HFs

**Figure 2.**
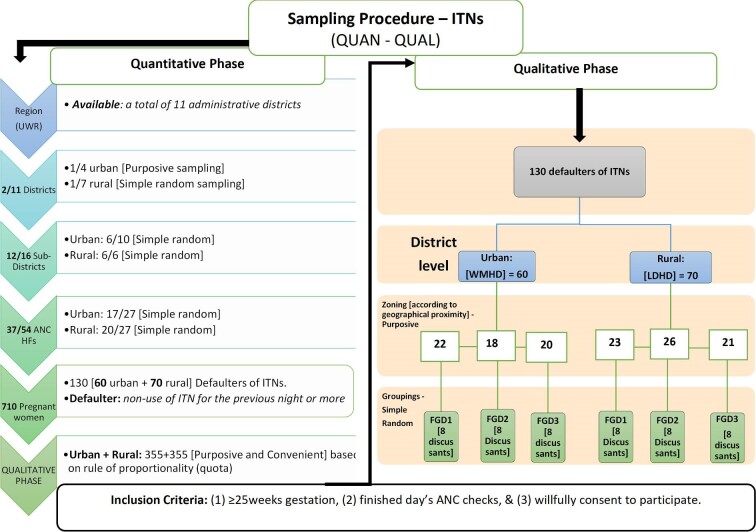
Study design and sampling procedure for ITN ownership and use.

A multistage sampling approach was used (Figure 2).^15^ Two of 11 administrative districts in the UWR (1 urban [purposive] and 1 rural [simple random]) were selected for comparison (Figure 1).^18^ The oldest, most populated and comparatively more resourced of the four urban districts was purposefully selected and compared with one of seven rural districts that was randomly selected. Unlike the urban district, the rural district is underresourced, with dispersed population density and often lacking the requisite health staff. There were 27 HFs offering ANC services in the selected rural district of Lambussie and 27 in the selected urban district of Wa. Through a mix of purposive and simple random sampling, we selected 20 of 27 HFs in Lambussie and 17 of 27 HFs in Wa, for a total of 37 of 54 HFs from both districts. The main HF in each subdistrict, usually the highest referral centre, was automatically included in the sampled HFs; all other HFs were selected through simple random sampling (Table 5, Appendix 1). Prior to the random selection of the HFs, we adopted the simple majority rule of sampling 50%+1 of all eligible HFs in each subdistrict, as used elsewhere.^17,19^ Thus we sampled at least 50% of the total eligible HFs in each subdistrict. The 50%+1 rule was used because it was not feasible, due to time and other resource requirements, to cover all HFs in all selected subdistricts.^17^ Based on the total number of eligible HFs in each of the six subdistricts, the sum of 50%+1 of all eligible HFs added up to 20 and 17 HFs for the rural and urban districts, respectively (Table 6, Appendix 1).^17^

### Selection of study participants (pregnant women)

The pregnant women were selected from the chosen ANC facilities if they met the inclusion criteria (Figure 2). The pregnant women were recruited first by sequential sampling and then by purposive sampling (at least 25 weeks pregnant). The number of participants sampled from each health facility was based on the rule of proportionality (Table 7, Appendix 1).

#### Standard questionnaire

A semi-structured questionnaire was used by 12 study nurses. The series of questions asked about respondents’ knowledge of malaria and its prevention, the risks of malaria in pregnancy, history of ANC contacts, ownership and use of ITNs, current gestational age and at first ANC visit, parity and sociodemographic characteristics.

### Qualitative data

#### FGDs with pregnant women

The FGDs used an interactive question-and-answer format. The sample frame for the FGDs comprised 130 (60 urban and 70 rural) ‘ITN defaulters’ identified through the survey. An ITN defaulter was defined as any pregnant woman who had not slept under an ITN during the previous night (Figure 2).

Six FGDs with eight participants each were conducted.^20,21^ The participants for the FGDs in each district were drawn through a purposive mixed method (only defaulters) and a simple random (lottery) technique (if there were more than eight).^22^ All women were contacted via phone calls in collaboration with the responsible health workers and invited to participate voluntarily. The main language of communication throughout all FGDs was Dagaare, the primary traditional language spoken in the study area.

Data were collected through an FGD guide, an audio recorder and note pads. The FGD guide was developed based on recommended scientific standards.^22–24^ The guide consisted of questions sectioned into four main themes: participants’ basic understanding of malaria and its associated risks on pregnancy, challenges in accessing or using an ITN, if spouses or other family members posed any hindrance to their use of an ITN and if they felt that the service providers should consider facilitating their access to and use of ITNs.

### Data analyses

#### Quantitative data

Quantitative data were compiled, cleaned and analysed using Stata (version 14.0; StataCorp, College Station, TX, USA). After descriptive statistics, binary logistic regression was used to analyse the determinants of ITN use. The outcome variable was sleeping under an ITN, measured as categorical (yes/no). The independent variables measured as categorical included marital status, family type, occupation, monthly income, religion, formal education, level of education, gestation at first ANC, parity, ITN ownership, knowledge of risks of malaria in pregnancy and study district. Other variables measured as continuous included the number of ANC contacts, household size and maternal age.

#### Qualitative data

Thematic content analysis was used to analyse the qualitative data, considering both inductive and deductive approaches using the QDA Miner Lite and MS Word (Microsoft, Redmond, WA, USA). The recordings of the FGD were translated into English shortly after each group session. The audio recordings were translated independently by the two moderators of the FGDs and compared for validation and analysis. The recordings were played repeatedly and typed verbatim into MS Word according to the flow of the questioning and answering format. The text was validated by repeated reading alongside playing the recordings to ensure that no information was skipped. The responses and discussions for each question were tabulated into similar themes and synthesized according to inductive codes. The emerging expressions were identified and used as inductive codes for the respective themes.

## Results

### Quantitative data

Table 1 presents the sociodemographic characteristics of the 664 study participants (339 from the urban district and 325 from the rural district). The median age of the pregnant women was 26 y (standard deviation 5.8). The majority were petty traders (63.4%) and married (93.0%). One-third had no formal education, while 18.1% had a tertiary-level education. Two-thirds were Muslims and one-third were Christians. A total of 89% owned an ITN and 78% reported using it. ITN use was defined as a pregnant woman having slept under an ITN the previous night. There were no major differences between the rural and urban study populations (Table 1).

**Table 1.** Pregnant women's sociodemographic characteristics

	Full sample (N=664)		Urban (n=339)		Rural (n=325)	
Variable	n	%	n	%	n	%
Age group (years)						
15–20	81	12.2	35	10.3	46	14.2
21–25	219	33.0	118	34.8	101	31.1
26–30	179	27.0	103	30.4	76	23.4
31–35	136	20.5	63	18.7	73	22.5
36–40	27	4.1	16	4.7	11	3.4
41–45	22	3.3	4	1.1	18	5.5
Occupational status						
Farming	75	11.3	7	2.1	69	20.2
Public service	48	7.2	33	9.7	14	4.3
Petty trading	420	63.3	216	63.7	204	62.8
Unemployed	121	18.2	83	24.5	38	11.7
Marital status						
Married	619	93.2	328	96.7	291	89.5
Single	45	6.8	11	3.3	34	10.5
Family category (married only)						
Polygamous	186	30.0	77	23.4	109	33.5
Monogamous	433	70.0	262	79.9	216	66.5
Religious affiliation						
Muslim	431	64.9	260	76.7	172	52.9
Christian	212	31.9	79	23.3	132	40.6
ATR	21	3.2	0	0.0	21	6.5
Formal education						
Primary/none	239	36.0	102	30.1	136	41.8
Junior high	78	11.7	53	15.6	27	8.3
Senior high	226	34.0	115	33.9	111	34.2
Tertiary	121	18.2	69	20.4	51	15.7
Household size (persons)						
1–3	268	40.4	143	42.2	125	38.4
4–6	288	43.3	141	41.6	147	45.2
>6	108	16.3	55	16.2	53	16.3
Own ITN						
No	73	11.0	44	13.0	29	8.9
Yes	591	89.0	295	87.0	296	91.1
Trimester-based ownership of ITNs (weeks; yes only)						
First (≤12 weeks)	193	32.7	89	30.2	104	35.1
Second (>12–≤24 weeks)	363	61.4	189	64.0	174	58.8
Third (≥25 weeks)	35	5.9	17	5.8	18	6.1
Own and use ITN^a^						
No	130	22.0	60	20.4	70	23.7
Yes	461	78.0	235	79.6	226	76.3
Total	664	100.0	339	100.0	325	100.0

^a^ITN use: if a respondent slept under an ITN the previous night.

The obstetric and gynaecological characteristics of the respondents are presented in Table 2. The results show that the majority (57.3%) of respondents in the rural area were multigravida women, compared with 38.0% in the urban area. The majority of pregnancies had a gestation of 25–30 weeks; this was similar across the rural (56.4%) and urban (59.8%) areas. A total of 77% had between 4 and 7 ANC contacts.

**Table 2.** Pregnant women's obstetrical and gynaecological characteristics

Variable	Full sample (N=664)		Urban (n=339)		Rural (n=325)	
	n	%	n	%	n	%
Gravidity						
Primidgravida	167	25.2	106	31.3	61	18.8
Secundigravida	184	27.7	109	32.2	75	23.0
Multigravida	313	47.1	124	36.5	189	58.2
Gestational age at first ANC visit (weeks)						
≤12	207	31.1	96	28.3	111	34.2
>12–≤24	416	62.7	221	65.2	195	60.0
≥25	41	6.2	22	6.5	19	5.8
Gestational age on the day of the interview (weeks)						
25–30	386	58.1	203	59.9	183	56.3
31–36	219	33.0	108	31.9	111	34.2
37–42	59	8.9	28	8.2	31	9.5
Parity						
Nulliparous	174	26.2	110	32.4	63	19.7
Primiparous	164	24.7	100	29.5	64	19.8
Multiparous	311	46.8	126	37.2	185	57.0
Grand multiparous (≥5)	15	2.3	3	0.9	12	3.5
ANC contacts						
1	24	3.6	10	2.4	13	4.0
2	23	3.4	13	3.8	10	3.1
3	92	13.9	56	16.5	37	11.4
4	183	27.6	89	26.3	94	28.9
5	120	18.1	63	18.6	57	17.5
6	101	15.2	56	16.5	45	13.8
7	107	16.1	47	13.9	60	18.5
8	14	2.1	5	1.5	9	2.8

Table 3 depicts the respondents’ knowledge of the risks of malaria in pregnancy, with a mean knowledge score. The mean knowledge score indicates the proportion of general knowledge on the respective questions about malaria and its prevention. In all, 67.3% of respondents had a general knowledge of malaria in pregnancy, but with large variations for the specific questions. Notably, only 20.0% knew how malaria could affect the unborn child, with twice the number of rural pregnant women being knowledgeable than their urban counterparts. Knowledge characteristics across urban and rural areas were relatively the same.

**Table 3.** Knowledge of malaria in pregnancy among pregnant women (correct responses only)

	Full sample (N=664)		Urban N=339		Rural N=325	
Knowledge parameter	n	%	n	%	n	%
Main cause of malaria	549	82.7	273	80.5	276	84.9
Main methods used to protect yourself against malaria	641	96.5	329	97.1	312	96.0
Malaria in pregnancy can harm your unborn child	422	63.6	194	57.2	228	70.2
Ways malaria can harm you and/or your unborn baby	133	20.0	43	12.7	90	27.7
The first SP is enough to protect you throughout pregnancy	198	29.8	72	21.2	126	38.8
Only sick (of malaria) pregnant women need to use an ITN	566	85.2	277	81.7	289	88.9
The SP therapy harms your unborn child	622	93.7	308	90.9	314	96.6
Mean knowledge score	447	67.3	214	63.1	234	72.0

SP: sulfadoxine-pyrimethamine.

Table 4 shows the binary logistic regression of sociodemographic characteristics of respondents on the use of ITNs. The results show that the main determinants of the use of ITNs include owning an ITN (odds ratio [OR] 2.4 [95% confidence interval {CI} 1.3 to 4.4]), good maternal knowledge of the risks of malaria in pregnancy (OR 2.4 [95% CI 1.3 to 4.3]) and more ANC contacts (OR 1.3 [95% CI 1.0 to 1.5]). The character of gestational age at first ANC registration varies according to the trimester: both third- (OR 4.7 [95% CI 1.5 to 15.2]) and second-trimester women (OR 3.5 [95% CI 2.0 to 6.1]) had a statistically significant positive influence on ITN use compared with women in the first trimester. Religious affiliations (Muslim OR 0.6 [95% CI 0.4 to 1.0] and African traditional religion [ATR] OR 0.3 [95% CI 0.1 to 0.9]) also showed a statistically significant negative influence on ITN use compared with Christianity, as well as being married compared with not being married (OR 0.3 [95% CI 0.1 to 0.6]). However, there was no statistically significant influence of living in a rural or urban environment on ITN use.

**Table 4.** Binary logistic regression on the use of ITNs

Use of ITN (yes/no)^a^	Subcategories	OR	95% CI		p-Value*
Age		1.0	0.9	1.1	NS
Marital status	Married	ref			
	Single	0.3	0.1	0.6	0.001
Family type	Polygamous	ref			
	Monogamous	1.1	0.7	1.8	NS
	Not married	1.4	0.1	21.95	NS
Occupation	Farming	ref			
	Public/civil service	0.7	0.3	2.0	NS
	Private/personal business	1.8	0.9	3.9	NS
	Unemployed	1.9	0.7	4.1	NS
Monthly income	Below poverty line (GHS 0–299)	ref			
	Within poverty line (GHS 300–599)	0.8	0.3	2.3	NS
	Above poverty line (GHS 600–1800)	0.7	0.2	1.9	NS
Religion	Christian	ref			
	Muslim	0.6	0.3	1.0	0.039
	ATR	0.3	0.1	0.9	0.037
Formal education	Yes	ref			
	No	1.4	0.5	4.2	NS
Level of education	Primary	ref			
	Junior high	1.0	0.3	3.1	NS
	Senior high	3.1	1.0	9.7	NS
	Tertiary	1.6	0.5	5.2	NS
Household size		1.0	1.0	1.2	NS
Gestational age at first ANC	1st trimester	ref			
	2nd trimester	3.5	2.0	6.1	0.001
	3rd trimester	4.7	1.5	15.2	0.010
Number of ANC contacts		1.3	1.0	1.5	0.018
Parity	Nulliparous	ref			
	Primiparous	0.6	0.3	1.1	NS
	Para 2–3	1.0	0.5	1.7	NS
	Multiparous (4–8)	0.4	0.2	1.1	NS
Own an ITN	No	ref			
	Yes	2.4	1.3	4.4	0.005
Knowledge of malaria in pregnancy	Poor	ref			
	Good	2.4	1.3	4.3	0.004
Study district	Urban	ref			
	Rural	0.8	0.5	1.2	NS

GHS: Ghana cedis; NS: not significant.

*Statistically significant at p<0.05.

^a^Use of ITN was defined as having slept under an ITN the previous night or more.

### Qualitative data

#### General knowledge about malaria

The information from the FGDs shows that pregnant women generally had good knowledge about the cause and prevention of malaria. Regarding the cause of malaria, the factor of poor environmental conditions such as ‘stagnant waters…’, show that respondents were aware of how specific environmental conditions can support the survival of the malaria vector. Others also precisely identified the ‘bites from a mosquito’ and ‘the use of a net [ITN],’ ‘insecticide sprays’ or ‘repellents’ as the cause and prevention of malaria, respectively. However, others felt that malaria is caused by ‘eating cold food or drinking cold water’, or even ‘cold weather’.

#### Challenges to the use of ITNs among pregnant women

**Figure 3.**
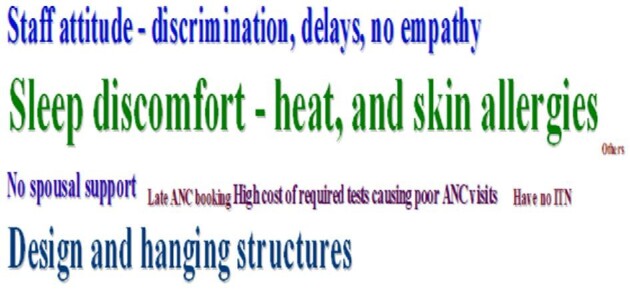
Challenges for regular use of ITN mentioned in FGDs.

Figure 3 shows the challenges to the regular use of ITNs among pregnant women. The most frequent reason for non-regular use of ITNs was the ‘sleep discomfort of heat, skin itching, and rashes’:

‘For me, I get facial rashes and itches anytime I sleep inside the mosquito net because of the chemical used to spray the nets. It will be good also if they make the mosquito nets more air-friendly, I don't know how that can be done, but that will make it better for us to sleep in it’. — Participant, FGD1_WMHD (Table 8, Appendix 1).

‘The net inside? I can't sleep inside; it is because of the heat’. — Participant, FGD3_WMHD

Pregnant women also complained about the lack of or inappropriate hanging structures in their homes to hang the nets over their beds. They explained this would be less problematic if all the ITNs were designed in a conical shape with one hanging rope rather than the rectangular shape with four ropes:

‘Some of us, how to lay is always the problem, we have to use a lot of nails to pin on the walls before we can lay it, but most landlords don't allow us to use nails on their walls, so that prevents us from using’. — Participant, FGD3_WMHD (Table 8, Appendix 1).

Another pronounced challenge was

‘insufficient or lack of spousal support’. — Participant, FGD3_WMHD (Figure 3)

#### Ways to facilitate regular use of ITNs

**Figure 4.**
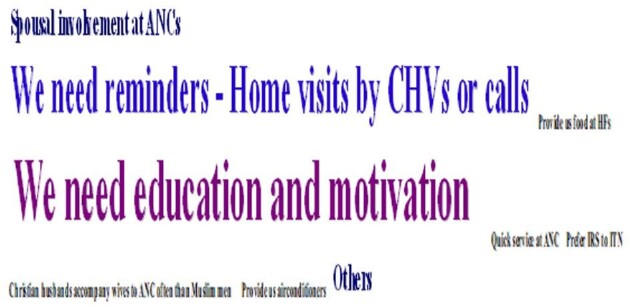
Suggestions to improve adherence to ITN use.

Of the many needs expressed during the discussion of this theme (Figure 4), the most dominant was their need for more ‘education and encouragement from service providers’, as beautifully put by some participants:

‘For me, motivation from the nurses, education on the importance of it, telling us more on the consequences of sleeping outside the mosquito net will help us’. — Participant, FGD2_LDHD (Table 7, Appendix 1).

‘They should be motivating us, boxing [cajoling] us, especially whenever we get to the weighing ground [referring to the ANC] at the facility. It will encourage us to use the ITN…’. — Participant, FGD1_LDHD (Table 7, Appendix 1).

### Discussion

The regular and proper use of ITNs is one of the most effective ways to avoid mosquito bites and contracting malaria.^25,26^ Unlike findings reported in similar studies elsewhere,^27^ the majority (78%) of our study respondents who owned ITNs (89%) also reported using them.^3,28,29^ However, unlike our study, which was facility-based, other studies were frequently household surveys.^28,29^ Therefore our findings could be partly attributed to response bias. ITNs are usually freely distributed at ANC services to all registered pregnant women. However, our findings show that fewer pregnant women received ITNs in their first trimester than in the second trimester. This could be a possible reason for the low ITN use in the first trimester, since ITN ownership also statistically influenced ITN use in this study. Our study revealed that ownership of an ITN, good maternal knowledge of the risks of malaria in pregnancy, more ANC visits and the gestational age at the mother's first ANC visit are important determinants of the regular and proper use of ITNs in the study area.

Our finding of a significant relationship between specific knowledge of the risks of malaria in pregnancy and the use of ITNs is similar to findings of related studies.^28,30,31^ However, these studies assessed the general knowledge of malaria, unlike our study. In terms of maternal educational background, our study did not find any significant influence on ITN use, as reflected in similar studies by Oladimeji et al.^27^ and Adebayo et al.,^30^ but contrary to reports in the studies of Oladimeji et al.^27^ and the WHO.^32^ This shows that behaviour change is not just influenced by knowledge per se or formal education, but by knowledge very specific to the target audience's needs.^30^ Gestational age at the first ANC and more ANC contacts also showed a significant relationship with the use of ITNs. This is not surprising, because ITNs are often distributed freely to pregnant women at ANC facilities upon registration or during ANC visits.^25,33^ In this finding, however, pregnant women who registered at an ANC facility in their second or third trimester were about five times and three times more likely to use their ITNs, respectively, than those who registered for ANC during their first trimester. This finding is rather surprising, and the plausible reason could be the timing when the ITN was given: if the pregnant women received her ITN in the first trimester, it is likely that they would forget the importance of sleeping under it until later in their pregnancy. This emphasizes the need for repetitive health education at every ANC contact throughout the pregnancy. Our findings of a significant relationship between gestation at registration, more ANC visits and ITN use support the findings of related studies elsewhere.^3,25,28^

Our study also revealed that marital status was associated with the regular use of ITNs. Single pregnant women were 0.3 times less likely to use their ITNs compared with married pregnant women. This finding supports similar reports of other studies.^25,27,32^ This could be because the married women possibly benefit from spousal support, unlike single women, who may have to manage pregnancy-related and resource-demanding needs alone. Maternal religious affiliation also played a role in the respondents’ decision to use ITNs. Both ATR-affiliated and Muslim pregnant women were less likely (0.3 and 0.6, respectively) to use ITNs than Christian pregnant women. The findings of religion as a possible determinant of ITN use reflect findings of another study in northern Ghana^34^ but contradict findings of a related study in Uganda.^3^ Our finding of a reduced likelihood of ITN use among ATR and Muslim women compared with their Christian counterparts was affirmed in our FGD that ‘Muslim men are less supportive to their spouses’. Similar to other studies, we did not find any significant difference between the rural/urban residence of pregnant women and their use of ITNs,^27^ possibly indicating that access to ITNs is equal in both study districts. However, a related study in some parts of northern Ghana showed a difference between rural and urban areas.^34^

The purpose of the FGDs was to identify and understand the challenges to access and utilization of ITNs from the perspective of pregnant women. From the results, the most outstanding challenge to regular use of ITNs was the experience of ‘sleep discomfort’. In an almost unanimous voice, the pregnant women mentioned that they experience discomfort such as ‘heat or the feeling of excessive warm’ and ‘skin rashes’, among others, when using the ITNs. Other pronounced challenges included the ‘lack of or inappropriate hanging structures’ to mount their ITNs. Other studies on the topic have reported similar reasons.^9,30,32–36^

## Conclusions

Adequate maternal knowledge of malaria risks in pregnancy is strongly related to regular ITN use among pregnant women in northern Ghana. Health policymakers in Ghana and similar settings in SSA need to formulate community- and facility-level interventions that encourage knowledge acquisition of the risks of malaria in pregnancy. Additionally, more user-friendly ITN types should be made available to communities in malaria-endemic areas.

## Data Availability

All data are available to the readers in this article. However, in the event that details are required, inquiries can be made to the corresponding author.

## Appendix 1

**Table 5.** Distribution of study districts, subdistricts and ANC HFs selected

Region	Districts	Subdistricts selected	HFs available	HFs selected
Ghana/Upper Wes Region	Wa Municipal (Urban)	Bamahu	7	4
		Busa	3	2
		Charia	2	1
		Wa North	6	4
		Wa South	5	3
		Kambali	4	3
		Subtotal	27	17
	Lambussie District (Rural)	Billaw	5	3
		Hamile	7	6
		Karne	4	2
		Lambussie Main	1	1
		Piina	3	3
		Samoa	7	5
		Subtotal	27	20
Total	2/11	12/16	54	37

**Table 6.** Proportions of pregnant women recruited in each subdistrict in the two selected districts

District	Year	Subdistrict	ANC population (A)	Percentage of total ANC (B)	Sample size quotas
					ITNs (Q)=(B/100)×A
Wa Municipality	2018 ANC register	Bamahu	549	10.4	37
		Busa	290	5.5	19
		Charia	175	3.4	12
		Wa North	861	16.3	58
		Wa South	2446	46.4	165
		Kambali	949	18.0	64
		Sub-total (N1)	5270	100.0	355
Lambussie-Karni					
		Billaw	187	12.7	45
		Hamile	475	32.3	114
		Karni	235	16.0	57
		Lambussie	120	8.1	29
		Piina	169	11.5	41
		Samoa	286	19.4	69
		Subtotal (N2)	1472	100.0	355
		Total (N)	7968	100.0	710

%B: the percentage of the subtotal ANC population (N1 or N2) a particular subdistrict constitutes.

Demonstration: for the Bamahu subdistrict, research assistants sampled 37 of the 549 registered pregnant women, computed as follows: {(A/N1)×100}=B%. A (for the Bamahu subdistrict)=549 and N1=5270. Thus {(549/5270)×100}=10.4%. 10.4% of 549=37 (respondents).

Total (N)=N1+N2.

**Table 7.** Proportions of pregnant women recruited at HFs in the two selected districts

				ITNs use (yes)	
Study district	HF	n	%	n	%
Urban (Wa Municipal)	Bamahu HC	19	5.6	11	57.9
	Danko CHPS	6	1.8	5	83.3
	Piisi CHPS	1	0.3	1	100.0
	UDS Hospital	6	1.8	3	50.0
	Busa HC	14	4.1	9	64.3
	Jonga CHPS	5	1.5	4	80.0
	Charia HC	11	3.2	8	72.7
	Kambali HC	48	14.1	22	45.8
	Mangu CHPS	5	1.5	4	80.0
	Nakori CHPS	5	1.5	2	40.0
	Market Clinic	18	5.3	11	61.1
	Adolescent HC	3	0.9	2	66.7
	Fongu CHPS	12	3.5	9	75.0
	Kumbiehi CHPS	14	4.1	10	71.4
	Wa Urban HC	144	42.4	113	78.5
	Sokpayiri CHPS	20	5.9	14	70.0
	Konta CHPS	8	2.4	7	87.5
	Total (N1)	339	100.0	235	69.3
Rural (Lambussie)	Billaw HC	15	4.6	10	66.7
	Chebogo CHPS	11	3.4	9	81.8
	Hinenteng CHPS	13	4.0	12	92.3
	Hamili HC	36	11.1	24	66.7
	Happa CHPS	7	2.1	4	57.1
	Muslim Clinic	8	2.5	6	75.0
	Kanyiri Maternity Home	35	10.8	23	65.7
	Chetu CHPS	12	3.7	9	75.0
	Bamwon CHPS	3	0.9	0	0.0
	Karni HC	23	7.1	16	69.6
	Kpare CHPS	15	4.6	12	80.0
	Lambussie Polyclinic	57	17.5	37	64.9
	Piina HC	23	7.1	15	65.2
	Sentu CHPS	11	3.4	8	72.7
	Hacha CHPS	1	0.3	1	100.0
	Samoa HC	16	4.9	12	75.0
	Naawie CHPS	5	1.5	4	80.0
	Suke CHPS	13	4.0	9	69.2
	Chognuor CHPS	6	1.9	5	83.3
	Koro CHPS	15	4.6	10	66.7
	Total (N2)	325	100.0	226	69.5
Total (N1+N2)		664	100.0	461	69.4

CHPS: Community Health Planning and Services; HC: Health Centre; N1: total sample for rural; N2: total sample for urban.

**Table 8.** Quotes from FGDs on ITNs use: urban (WMHD) and rural (LDHD) districts

Deductive code/Theme	Verbatim Quotes	Inductive code
Basic understanding of malaria	‘All pregnant women already have malaria in them, still with that we worry to prevent malaria from affecting the child’. Participant, FGD2_WMHD*	Sound basic knowledge
	‘It is the mosquitos that give malaria, and that's only when you expose yourself to the mosquitoes by not sleeping under mosquito nets. Sometimes too, switching from bathing of cold and warm water can give you malaria. If you are also eating cold things or food, you can get malaria through that’. Participant, FGD_WMHD	
	‘Malaria comes from the mosquitoes, and the stagnant waters, overcrowded areas, dirty gutters in which there is stagnant water, stagnant waters behind bathrooms. The hanging of clothes on crossbars can breeds mosquitoes. Where there is a lot of water and mosquitoes are hake. When malaria gets you can be shivering, be dizziness, feeling cold, body weakness and dullness, general body pains and low appetite’. Participant, FGD3_WMHD	
	‘I know that as a pregnant woman, it is riskier getting malaria because I am not alone. They are some people when they get malaria during the pregnancy they cannot deliver. If you get malaria, you may struggle to give birth or risk complications during birth, so it is right that you protect yourself against malaria during pregnancy. It may even also get the unborn child’. Participant, FGD1_LDHD*	
	‘We can prevent and protect ourselves from mosquito bites by sleeping in mosquito nets, taking care of the food we eat during pregnancy. Taking care of your environment.’ Participant, FGD2_LDHD	
Challenges in accessing and/or using ITNs	‘It is the heat: when you, the pregnant woman, sleeps on it, it is mostly like you are suffocating and about to die; it limits the breathing ability. I know my body produces heat, and the heat from the mosquito nets also adds to it. I think it is because of the chemical they use to spray it’. Participant, FGD2_WMHD	Sleep discomforts
	‘For me, I do not know about it because I was not given a mosquito net. I was asked to go and do a test by the time I returned, the person who was distributing the ITNs had left.’ Participant, FGD2_LDHD	
	‘The net inside? I can't sleep inside; it is because of the heat’. Participant, FGD3_WMHD	
	‘For me, whenever I sleep under the ITN I get itches and body rashes, and I think it is the chemical they use to spray that itches my body’. Participant, FGD2_LDHD	
	‘For me, I get facial rashes and itches anytime I sleep inside the mosquito net, either because of the chemical used to spray the nets. It will be good also if they make the mosquito nets more air friendly, I don't know how that can be done, but that will make it better for us to sleep in it’. Participant, FGD1_WMHD	
Improving access to and interest in using ITNs	‘The major thing is the heat, we mentioned earlier, the holes of the mosquito nets are too tiny, and that prevents air from passing through it, also based on its name ‘mosquito net’ the holes cannot also be wider else we cannot protect ourselves against mosquitoes.’ Participant, FGD3_LDHD	
	‘Some of us, how to lay is always the problem, we have to use a lot of nails to pin on the walls before we can lay it, but most landlords don't allow us to use nails on their walls, so that prevent us from using.’ Participant, FGD3_WMHD	Structural challenge
	‘For me, motivation from the nurses, education on the importance of it, telling us more on the consequences of sleeping outside the mosquito net will help us.’ Participant, FGD2_LDHD	Education & Motivation
	‘How you can do, and there will not be heat when we are sleeping under it, will help. There is nothing to be done; we are already prone to heat as pregnant women.’ Participant, FGD2_WMHD	
	‘If it is only made of the single hanging robe, it will be better’. Participant, FGD3_WMHD	
	‘They should encouragingly talk to us, provide us with air conditions in our rooms so that we will not encounter heat in sleeping in the mosquito nets’. Participant. FGD1_WMHD	
	‘They should be motivating us, boxing [cajoling] us, etc., especially whenever we get to the weighing ground [referring to the ANC section] at the facility. It will encourage us to use the ITN and the SP.’ Participant, FGD1_LDHD	

LDHD: Lambussie District Health Directorate; WMHD: Wa Municipal Health Directorate.
